# Multimodal imaging of Hypotrichosis with juvenile macular dystrophy: a case report

**DOI:** 10.1186/s12886-021-02037-8

**Published:** 2021-07-23

**Authors:** Giovanna Carnovale-Scalzo, Adriano Carnevali, Gabriele Piccoli, Domenico Ceravolo, Donatella Bruzzichessi, Rodolfo Iuliano, Rossana Tallerico, Valentina Gatti, Giuseppe Giannaccare, Vincenzo Scorcia

**Affiliations:** 1 Department of Ophthalmology, University “Magna Græcia”, Viale Europa, Loc. Germaneto, Catanzaro, Italy; 2 Medical Genetics Unit, Clinical and Experimental Medicine Department, University of “Magna Græcia”, Catanzaro, Italy

**Keywords:** CH3 mutation, Juvenile macular dystrophy, MfERG, Optical coherence tomography angiography, Case report

## Abstract

**Background:**

To report the first Italian case of hypotrichosis with juvenile macular dystrophy complicated by macular neovascularization diagnosed through multimodal imaging.

**Case presentation:**

An 11-year-old boy was referred to our Institution for bilateral maculopathy of unknown origin. Multimodal imaging helps the diagnosis of Juvenile Macular Dystrophy with Hypotrichosis (HJMD). Fundus examination showed several alterations of the retinal pigment epithelium and circular pigmented area of chorioretinal atrophy. Structural spectral domain optical coherence tomography (OCT) showed some backscattering phenomenon with several alterations of retinal pigment epithelium and photoreceptor layer in both eyes. Moreover, OCT showed hyperreflective lesion beneath the neuroepithelium in left eye. OCT angiography (OCT-A) revealed a pathologic neovascular network in choriocapillaris plexus, probably the result of a fibrovascular membrane. Multifocal electroretinograms (MfERGs) showed functional alterations in 12.22° of the central retina. In order to confirm the suspicion of HJMD, the child and both parents underwent genetic testing. Both parents resulted to be heterozygous healthy carriers of a single variation.

**Conclusion:**

Multimodal imaging, in particular OCT-A, is a useful aid, along to clinical findings and genetics, for the diagnosis of inherited retinal dystrophies.

## Background

Hypotrichosis with juvenile macular dystrophy (HJMD) is a rare autosomal-recessive disorder characterized by progressive hair loss and macular degeneration. To date, about 50 cases have been described since the first report [1–4]. It is caused by mutations in the CDH3 (16q22.1) [5], which encodes for P-cadherin. The glycoprotein is part of the adhesion junctions of various epithelia, including the follicular one [6] and the retinal pigment epithelium (RPE) [7, 8]. Patients with HJMD present short and sparse hairs since birth that do not grow thereafter. Visual acuity decreases between the first and third decades of life and blindness can be experienced later. The diagnosis is clinical, based on the association of hypotrichosis and macular pigment abnormalities upon fundus examination. Genetic-molecular test of the CDH3 gene must be performed to reach a definite diagnosis.

We report the multimodal imaging, including Optical Coherence Tomography Angiography (OCT-A), of a rare case of HJMD occurring in an Italian child.

## Case presentation

This is a retrospective review of clinical data of a patient with hypotrichosis followed at University Magna Graecia of Catanzaro at the Department of Ophthalmology.

**Fig. 1:**
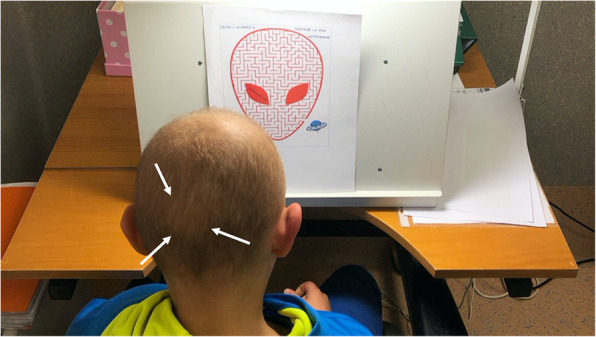
Representative image from the patient with sparse hair with hypotrichosis

An 11-year-old boy was referred to our Department for bilateral maculopathy of unknown origin. The child was born at 37th week by caesarean section, being the second born from unrelated parents. After the delivery, the child presented alopecia, roundish facies, hypotrichosis with light lanugo hairs (Fig. 1), sparse eyelashes, hypotelorism, small hands, atopic dermatitis. Ocular history was unremarkable until the age of 4 years when ophthalmological examination revealed an history of Stargardt-like maculopathy, parents reported that the onset of visual impairment occurred at the age of 8 years and that the child did not have history of amblyopia. Upon presentation, best-corrected visual acuity (BCVA) was 0.3 logMAR in right eye (RE) and 1 logMAR in left eye (LE). Nystagmus was not observed. The patient also had photophobia, decrease in color vision evaluated with the Farnsworth D-15 test, decrease in contrast sensitivity at eye chart REX (Reading Explorer) test and an increase in glare sensitivity investigated with Berkeley glare test.

**Fig. 2:**
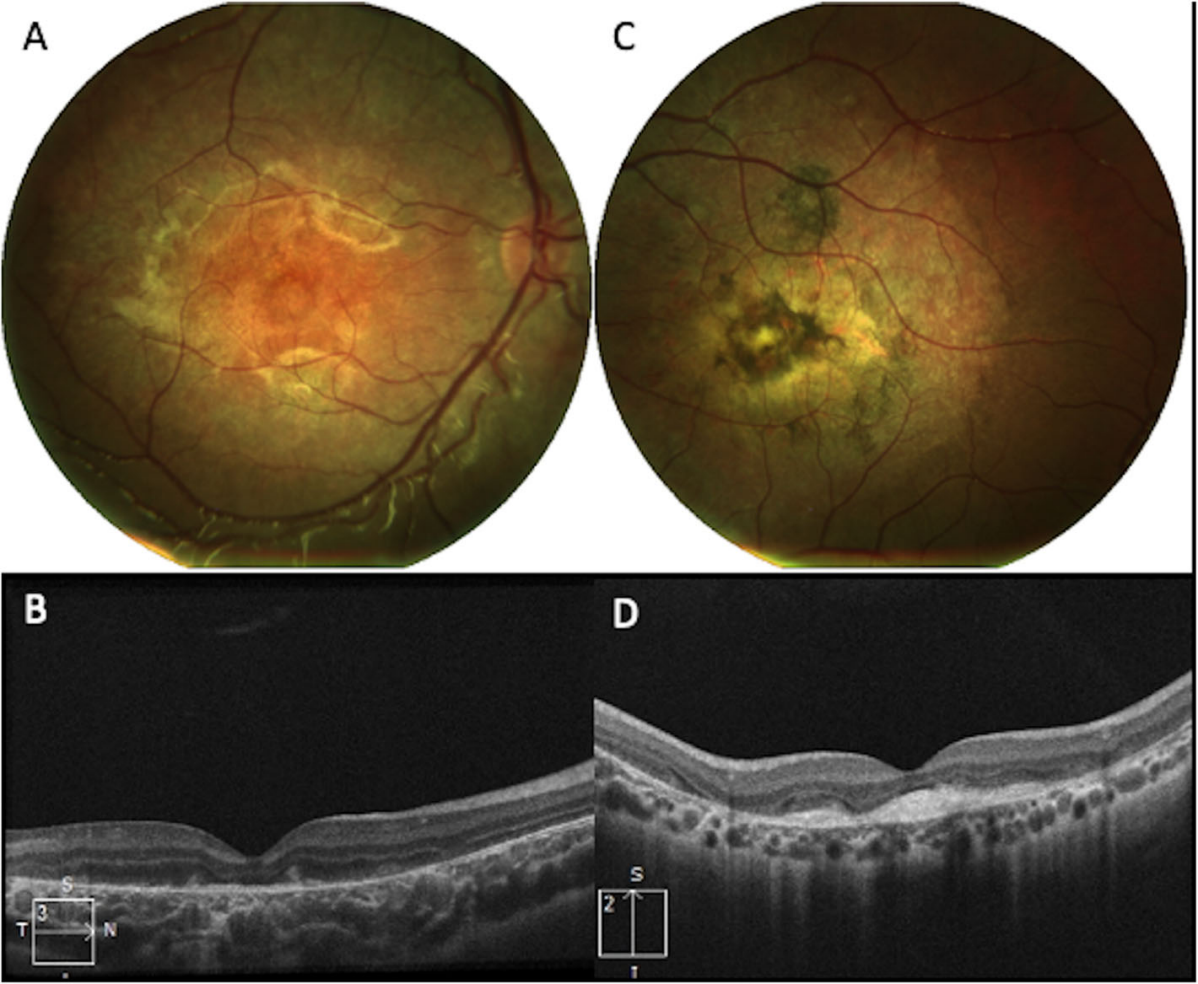
(A-B) Fundus photo of the right eye shows several alterations of the retinal pigment epithelium (RPE) with accentuation of the axial reflex (bull’s eye maculopathy). B-scan optical coherence tomography (OCT) shows some backscattering phenomenon with several alterations of RPE and photoreceptor layer. C-D) Fundus photo of the left eye revealed a circular pigmented area of chorioretinal atrophy; OCT shows several phenomena of back-scattering with hyperreflective lesion beneath the neuroepithelium

**Fig. 3:**
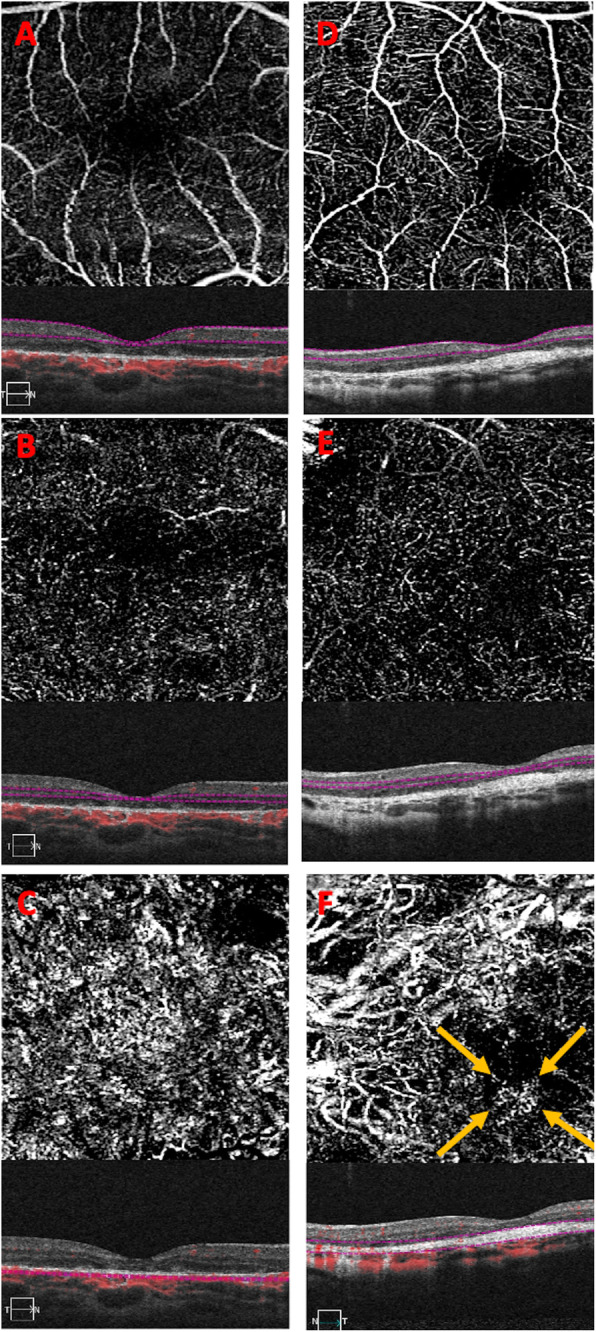
A-B-C) 3X3 mm optical coherence tomography angiography (OCT-A) of superficial, deep and choriocapillary plexuses and correlating optical coherence tomography (OCT) B-scans of the right eye. OCT-A revealed capillary rarefaction in superficial, deep and choriocapillary plexuses. D-E-F) 3X3 mm OCT-A of superficial, deep and choriocapillary plexuses and correlating OCT B-scans of the left eye. OCT-A revealed capillary rarefaction in superficial and deep plexuses, and a loss of choriocapillaris flow under the atrophic patches. Moreover, in correspondence of the hyperreflective lesion (visualized on B-scan OCT) OCT-A shows a pathologic neovascular network (yellow arrows), probably the result of a fibrovascular membrane

**Fig. 4:**
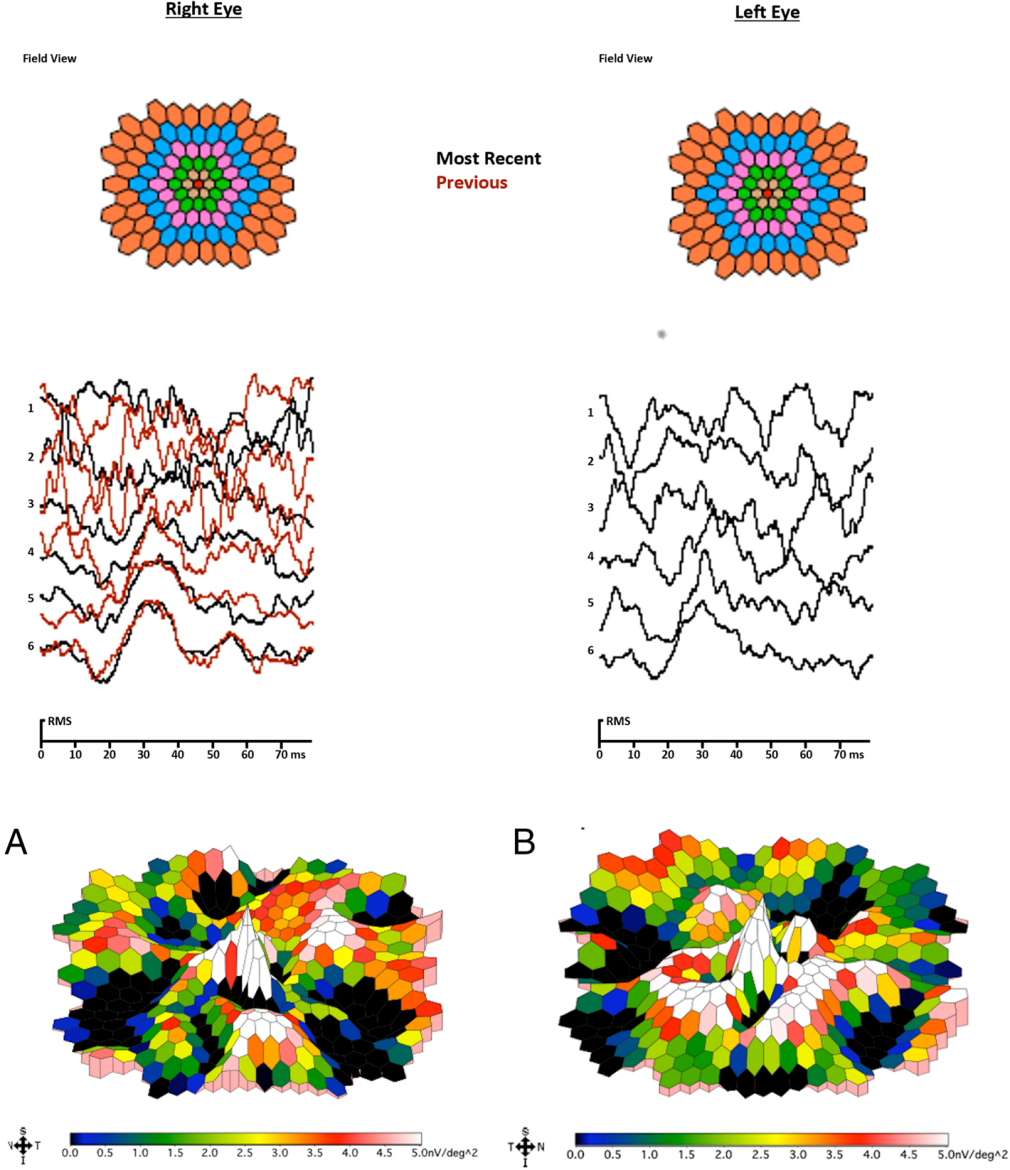
Electrophysiological test Multifocal electroretinogram mfERG. A. A&B Second Order Kernel - 3D plot scalar of the amplitudes of the MfERG shows functional alterations in 12.22° of the central retina

Stereoscopic acuity with Lang II sterotest measured 400″. Fundus photograph (Nidek MP-1, microperimeter and retinography) of RE showed several alterations of RPE with accentuation of the axial reflex (bull’s eye maculopathy). B-scan Spectral domain Optical Coherence Tomography (OCT) imaging (Zeiss Cirrus 5000-HD-OCT) showed backscattering phenomena with several alterations of RPE and photoreceptor layer. Fundus photo of LE revealed a circular pigmented area of chorioretinal atrophy while OCT showed phenomena of back-scattering with hyperreflective lesion beneath the neuroepithelium (Fig. 2). OCT-A (Zeiss Cirrus 5000-HD-OCT) 3X3 mm of superficial and deep plexuses revealed capillary rarefaction in both eyes. Instead OCT-A in choriocapillary plexus revealed capillary rarefaction in RE and a loss of choriocapillaris flow under the atrophic patches in LE. Moreover, in correspondence of the hyperreflective lesion (visualized on B-scan OCT in LE) OCT-A shows a pathologic neovascular network, likely the result of a fibrovascular membrane (Fig. 3). Multifocal electroretinograms (MfERGs) were performed on Visual Evoked Response Imaging System Science 6.0.5d5 unit according to International Society for Clinical Electrophysiology of Vision 2012 Standards [9]. The MfERG responses were divided into 6 concentric rings around the fovea from ring 1 (0°) to ring 6 (19.20°). Functional alterations were found in the 4th concentric ring (12.22°) of the central retina but not in the others (Fig. 4).

**Fig. 5:**
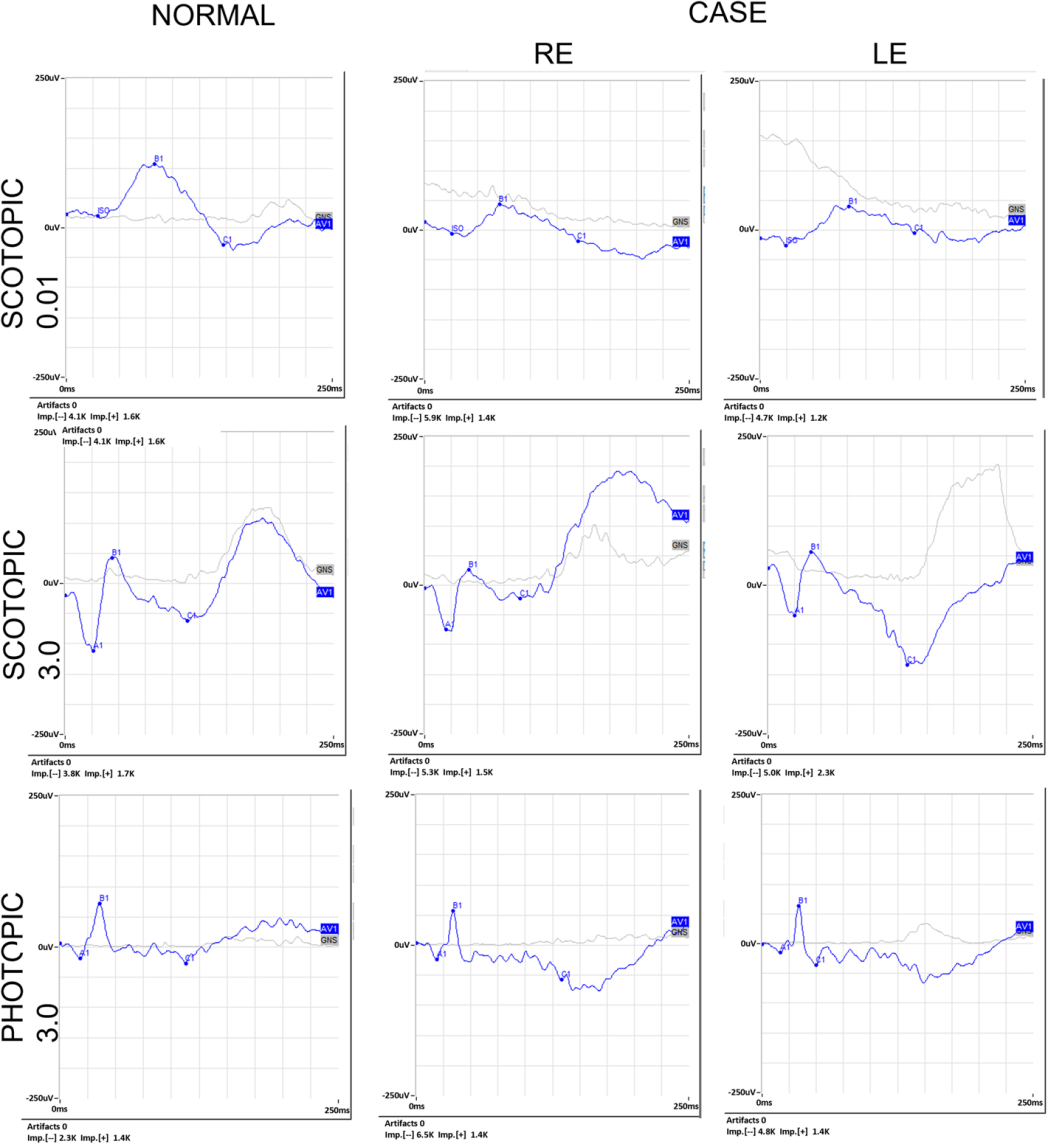
ERG recordings for both eyes, compared to control traces (left). Scotopic 0.01 ERG and combined rod- and cone dark-adapted standard 3.0 flash ERG show a severely reduced amplitude. Light-adapted single flash cone 3.0 ERG is normal in both eyes

In Fig. 5, we show the electroretinogram (ERG) results (Retimax, CSO, version 7.0.5). The scotopic 0.01 ERG showed a reduction in the amplitude of the b-wave in both eyes. The combined rod- and cone dark-adapted standard 3.0 flash ERG showed a negative ERG in both eyes with a reduction in the b-wave amplitude. The light-adapted single flash cone 3.0 ERG was normal in both eyes.

**Fig. 6:**
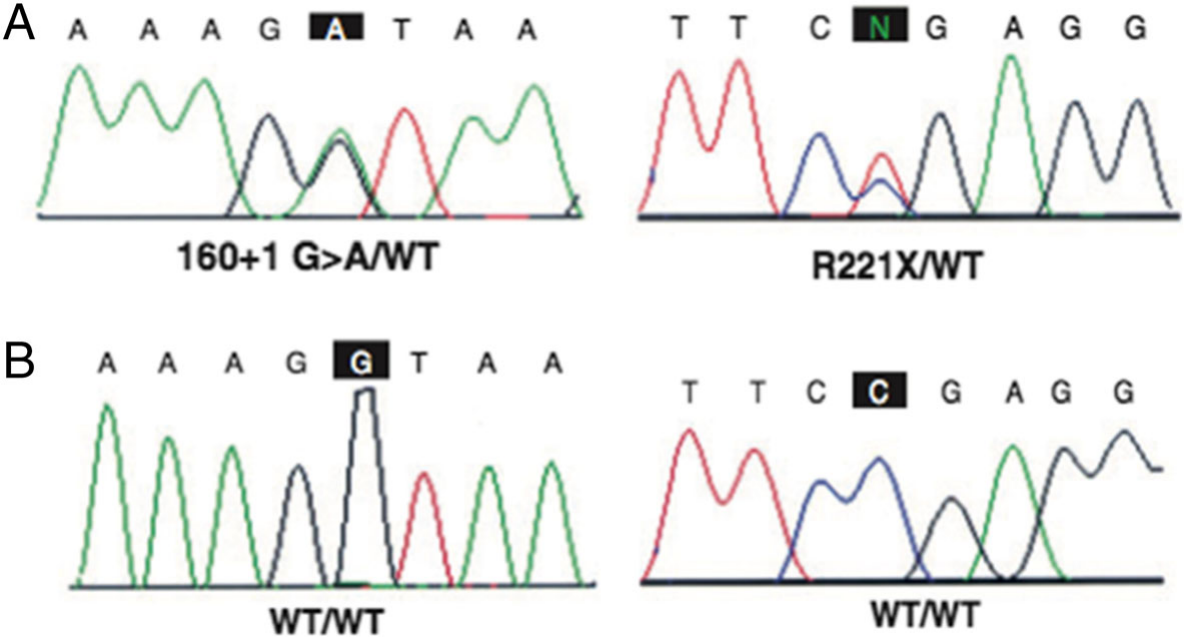
A-B) Electropherogram obtained by Sanger DNA sequencing, which shows the sequence with corresponding mutation present in the patient and respective wild-type sequence

In order to clarify the suspicion of HJMD, after a detailed explanation of the condition and the signature of informed consent for diagnostic procedures, the child and both parents underwent genetic testing. The analysis of all CDH3 exons with flanking intronic sequences detected in the child two variants, both in an apparent heterozygous state: the first one was a c.160 + 1G > A variant located at the first base in intron 2; the second one was a c.661C > T (p.R221X) variant, located at exon 6, already described to be causative for HJMD (Fig. 6) [10]. As expected, both parents resulted to be heterozygous healthy carriers of a single variation: the mother was positive for the c.160 + 1G > A variant while the father carried on the mutation.

## Discussion and conclusion

Nowadays, the syndrome HJMD has a prevalence of less than 1/1000000, according to Orphanet [11]. In all previous reports, reduced visual acuity was the first ocular symptom reported by patients and/or parents, as occurred also in our case. Usually, visual deterioration starts in the first decade of life [1, 4, 12–16]; however, Halford and co-authors described 2 patients who experienced visual loss later [17]. To date, a total number of 8 cases of HJMD have been described by means of OCT, fundus photography and MfERG. Here we report a rare case of the disease that was analyzed by a multimodal imaging also including OCT-A and confirmed by genetic testing. The c.661C > T mutation detected in our case has a frequency of 2/250996 alleles in the combined gnomAD Database (Genome Aggregated Database, Broad Institute, Cambridge, Massachusetts, USA) [18] and the consequence is a premature stop codon (p.R221X). In the present case, the onset of visual impairment occurred at the age of 8 years and worsened over the following years. However, at the last follow-up visit (age of 12 years), the visual acuity was 0,3 logMAR in RE and 1 logMAR in LE. The explanation for this different amount of vision between the two eyes is related to the more prominent disruption of the RPE and photoreceptor layers in both eyes with fibrovascular lesion in LE. The asymmetrical presentation of the disease is controversial with different hypotesis: several authors described symmetrical involvement of both eyes [1, 13, 17] and Indelman et colleagues [4] described an asymmetrical picture.

In our case, scotopic 0.01 ERG and combined rod and cone dark adapted standard 3.0 flash ERG showed reduced amplitude and MfERGs showed functional alterations in 12.22° of the central retina, which proves a cone-rod dysfunction as evidenced by Nasser F, et al. [19].

Macular Neovascularization (MNV) may occur in several chorioretinal diseases and has been reported rarely also in patients with inherited retinal dystrophies, accelerating visual loss [20]. In complex pathologies, OCT-A has provided novel insights into the relationship between flow index in MNV and density of the neovascular network [21–24]. In our patient OCT-A has been useful to confirm the presence of fibrovascular network without performing an invasive examination such as fluorescein angiography.

To our knowledge, our case has some characteristics that make it unique: the country of origin of the child (this is the first Italian case described) as well MNV in LE demonstrating with a multimodal imaging. Conversely, the limited information about long term anatomic and visual outcomes represents the main limitation of the present case report.

## Data Availability

All data generated or analysed during this study are included in this published article.

## Abbreviations

HJMD: Juvenile Macular Dystrophy with Hypotrichosis
RPE: Retinal Pigment Epithelium
OCT-A: Optical Coherence Tomography Angiography
BVCA: Best Visual Corrected Acuity
RE: Right Eye
LE: Left Eye
OCT: Optical Coherence Tomography
MfERGs: Multifocal electroretinograms
ERG: Electroretinogram
MNV: Macular Neovascularization

## References

[1] Khan AO, Bolz HJ (2016). Phenotypic observations in “hypotrichosis with juvenile macular dystrophy” (recessive CDH3 mutations). Ophthalmic Genet.

[2] Hull S, Arno G, Robson AG, Broadgate S, Plagnol V, McKibbin M (2016). Characterization of CDH3 Related Congenital Hypotrichosis With Juvenile Macular Dystrophy. JAMA Ophtalmol.

[3] Karti O, Abali S, Ayhan Z, Gokmeydan E, Nalcaci S, Yaman A, Saatci AO (2017). CDH3 gene related hypotrichosis and juvenile macular dystrophy - a case with a novel mutation. Am J Ophthalmol Case Rep.

[4] Indelman M, Eason J, Hummel M, Loza O, Suri M, Leys MJ, Bayne M, Schwartz FL, Sprecher E (2007). Novel CDH3 mutations in hypotrichosis with juvenile macular dystrophy. Clin Exp Dermatol.

[5] Sprecher E, Bergman R, Richard G, Lurie R, Shalev S, Petronius D, Shalata A, Anbinder Y, Leibu R, Perlman I, Cohen N, Szargel R (2001). Hypotrichosis with juvenile macular dystrophy is caused by a mutation in CDH3, encoding P-cadherin. Nat Genet.

[6] Shimoyama Y, Yoshida T, Terada M, Shimosato Y, Abe O, Hirohashi S (1989). Molecular cloning of a human Ca2+−dependent cell-cell adhesion molecule homologous to mouse placental cadherin: its low expression in human placental tissues. J Cell Biol.

[7] Burke JM, Cao F, Irving PE, Skumatz CM (1999). Expression of E-cadherin by human retinal pigment epithelium: delayed expression in vitro. Invest Ophthalmol Vis Sci.

[8] Shimomura Y, Wajid M, Shapiro L, Christiano AM (2008). P-cadherin is a p63 target gene with a crucial role in the developing human limb bud and hair follicle. Development.

[9] Hood CD, Bach M, Brigell M, Keating D, Kondo M, Lyons JS (2012). ISCEV standard for clinical multifocal electroretinography (mfERG) (2011 edition). Doc Ophthalmol.

[10] Lek M, Karczewski KJ, Minikel EV, Samocha KE, Banks E, Fennell T (2016). Analysis of protein-coding genetic variation in 60,706 humans. Nature.

[11] Orphanet. https://www.orpha.net/consor/cgi-bin/OC_Exp.php?lng=EN&Expert=1573#:~:text=Hypotrichosis%20with%20juvenile%20macular%20degeneration%20(HJMD)%20is%20a%20very%20rare,macular%20degeneration%20leading%20to%20blindness. Accessed 22 January 2021.

[12] Leibu R, Jermans A, Hatim G, Miller B, Sprecher E, Perlman I (2006). Hypotrichosis with juvenile macular dystrophy: clinical and electrophysiological assessment of visual function. Ophthalmology..

[13] Indelman M, Leibu R, Jammal A, Bergman R, Sprecher E (2005). Molecular basis of hypotrichosis with juvenile macular dystrophy in two siblings. Br J Dermatol.

[14] Fan KC, Patel NA, Yannuzzi NA, Prakhunhungsit S, Negron CI, Basora E, Colin AA, Tekin M, Berrocal AM (2019). A unique case of vision loss in a patient with hypotrichosis and juvenile macular dystrophy and primary ciliary dyskinesia. Am J Ophthalmol Case Rep.

[15] Narayan A, Moosajee M (2019). Sparse scalp hair and vision loss: think hypotrichosis with juvenile macular dystrophy. BMJ Case Rep.

[16] Ahmed A, Alali A, Alsharif O, Kaki A (2021). Hypotrichosis with juvenile macular dystrophy in Saudi Arabia: a case report. Skin Appendage Disord.

[17] Halford S, Holt R, Nemeth AH, Downes SM (2012). Homozygous deletion in CDH3 and hypotrichosis with juvenile macular dystrophy. Arch Ophthalmol.

[18] Karczewski KJ, Francioli LC, Tiao G, Cummings BB, Alföldi J, Wang Q (2020). The mutational constraint spectrum quantified from variation in 141,456 humans. Nature.

[19] Nasser F, Mulahasanovic L, Alkhateeb M, Biskup S, Stingl K, Zrenner E (2019). Hypotrichosis with cone-rod dystrophy in a patient with cadherin 3 (CDH3) mutation. Doc Ophthalmol.

[20] Marano F, Deutman AF, Leys A, Aandekerk AL (2000). Hereditary retinal dystrophies and choroidal neovascularization. Graefes Arch Clin Exp Ophthalmol.

[21] Carnevali A, Capuano V, Sacconi R, Querques L, Marchese A, Rabiolo A, Souied E, Scorcia V, Bandello F, Querques G (2017). OCT angiography of treatment-Naïve quiescent choroidal neovascularization in Pachychoroid Neovasculopathy. Ophthalmol Retina.

[22] Corbelli E, Carnevali A, Marchese A, Cicinelli MV, Querques L, Sacconi R, Bandello F, Querques G (2018). Optical coherence tomography angiography features of angioid streaks. Retina..

[23] Querques L, Parravano M, Borrelli E, Chiaravalloti A, Tedeschi M, Sacconi R, Zucchiatti I, Bandello F, Querques G (2020). Anatomical and functional changes in neovascular AMD in remission: comparison of fibrocellular and fibrovascular phenotypes. Br J Ophthalmol.

[24] Rabiolo A, Carnevali A, Bandello F, Querques G (2016). Optical coherence tomography angiography: evolution or revolution?. Expert Rev Ophthalmol.

