# Correction: Multimodal imaging of Hypotrichosis with juvenile macular dystrophy: a case report

**DOI:** 10.1186/s12886-024-03288-x

**Published:** 2024-01-19

**Authors:** Giovanna Carnovale-Scalzo, Adriano Carnevali, Gabriele Piccoli, Domenico Ceravolo, Donatella Bruzzichessi, Rodolfo Iuliano, Rossana Tallerico, Valentina Gatti, Giuseppe Giannaccare, Vincenzo Scorcia

**Affiliations:** 1 Department of Ophthalmology, University “Magna Graecia”, Viale Europa, Loc. Germaneto, Catanzaro, Italy; 2 Medical Genetics Unit, Clinical and Experimental Medicine Department, University of “Magna Graecia”, Catanzaro, Italy

**Correction:** ***BMC Ophthalmol*** **21, 284 (2021)**

10.1186/s12886-021-02037-8

In this article [1], the author name Giovanna Carnovale-Scalzo was incorrectly tagged in the XML version of the paper. The correct given name is Giovanna, and the family name Carnovale-Scalzo.

The author group has been updated above and the original article has been corrected.
